# “My skills are going to be exposed” – Anxiety, meaning and professional identity during simulation-based learning in medical students: A mixed method study

**DOI:** 10.1371/journal.pone.0327306

**Published:** 2025-07-09

**Authors:** Gareth Drake, Niki Skaltsa, Kritika Kalia, Chibueze Ogbonnaya, Asha Padhair, Samuel Morrish, Pratheeban Nambyiah

**Affiliations:** 1 Clinical Simulation Centre, Great Ormond Street Hospital Learning Academy, Great Ormond Street Children’s Hospital NHS Foundation Trust, London, United Kingdom; 2 West Suffolk Hospital NHS Foundation Trust, Bury Saint Edmunds, United Kingdom; 3 Berenberg Bank, London, United Kingdom; 4 Population, Policy & Practice Research & Teaching Department, Institute of Child Health, Faculty of Population Health Sciences, University College London, London, United Kingdom; 5 West Hertfordshire Hospitals NHS Trust, Hertfordshire, United Kingdom; 6 Sheffield Childrens Hospital NHS Foundation Trust, Sheffield, United Kingdom; Ankara University Faculty of Medicine: Ankara Universitesi Tip Fakultesi, TÜRKIYE

## Abstract

Clinical simulation is an established part of the educational offering for healthcare workers, yet for many it can induce feelings of anxiety and uncertainty. The role of these feelings in enhancing or impeding the pedagogical experience is unclear, but it is likely that there are mediating and moderating factors that affect the relationship. We undertook a prospective, observational, mixed methods study of a cohort of medical students undergoing a paediatric critical illness simulation course. Our quantitative aims were to understand whether increased state anxiety correlated negatively with simulation effectiveness. Our qualitative aim was to understand how students experienced their anxiety in relation to their learning experience during the simulation. We found that there was no significant relationship between state anxiety and simulation effectiveness. However, during qualitative interviews, we uncovered a rich seam of material regarding the students’ experience which coalesced around three broad themes – anticipation of the unknown, the nature of being observed, and the realism of clinical simulation, which elicited reflections among students on their future professional responsibilities and identity. We found that these feelings were simultaneously triggers for anxiety – and we suggest some practical ways of reducing the stress associated with this – and also the gateway to great insight for students into their learning process. Traditionally, learning in medicine has been associated with a maladaptive culture of perfection – high academic expectations, the perception that the ability to recall under pressure is the hallmark of a good doctor, and unwillingness to admit to vulnerability or error. Simulation leads students to the understanding that successful performance in real clinical settings requires asking for help, recognising the demands on and the limitations of available resources, and being able to navigate uncertainty.

## Introduction

Healthcare simulation involves the imitation of clinical events for the purposes of immersive education. It is common in medical training and has been shown to improve confidence, knowledge and skills [1]. Healthcare simulation typically comprises a pre-simulation briefing designed to cultivate psychological safety and orientate learners to the simulation environment [2]; a simulated clinical scenario or series of scenarios involving mannequins or actors (or, increasingly, extended reality modalities); and a post-simulation debrief for the purposes of consolidating learning and making links to clinical practice [3,4].

Despite the work that educators put into creating a safe learning environment, uncertainty, anxiety, and ambivalence remain among learners. Medical students have been found to display measurable stress responses during simulation [5,6]. A certain degree of stress can facilitate learning and memory formation [7,8], including during healthcare simulation [9]. One RCT has found that even the deeply unpleasant experience of shame facilitated learning among medical students under certain conditions [10]. Conversely, too much stress is counterproductive to learning and performance in healthcare simulation [11] and a pre-brief that decreased anxiety also improved confidence and performance [12].

The precise role that anxiety plays in enhancing or impeding pedagogical experience is unclear. Stein [13] found a complicated relationship between state anxiety – defined as temporary anxiety in response to a specific situation (cf. trait anxiety) – and performance in pre-hospital emergency care simulation performance. The relationship was described by a U-shaped curve, with improved performance at lower and higher state anxiety scores and reduced performance in the intermediate range. Lewis [14] found no relationship between pre-simulation state anxiety and performance in nurse anaesthesia students, but did find that perceived cognitive load was negatively correlated with performance. Medical student participants have reported that their simulation experience was negatively impacted by the “rollercoaster of emotions”, the act of role-playing, and by feeling uncertain whether to act as a medical student or as a qualified doctor. The difficulty they faced in stepping into an assumed role then made the students doubt their future competence [15]. One mixed methods study found that simulation learners with lower state anxiety scores rated simulation as more effective, reporting that a clear plan and expectations would have improved their experience, as would feeling less judged by faculty [16].

These complex findings suggest a role for mediating and moderating factors between anxiety and educational experience, potentially related to the ways in which individuals subjectively make sense of their states of mind in relation to their learning and professional development. The increased vulnerability among current healthcare workers [17] and students [18], coupled with increasing demand on services in the wake of the COVID-19 pandemic, means that it is now especially pertinent to seek to understand the factors that promote or impede learning and development in healthcare. Great Ormond Street Hospital (GOSH) for Children, where this research was undertaken, is a specialist UK children’s hospital. We have regular rotations of fifth-year medical students, who undertake a simulation course called Essential Paediatrics. This course trains students to manage common emergencies in paediatrics, and features many of the same demands that students might encounter in a real clinical setting. We aimed to examine what made it easier or more difficult for students attending this course to have a meaningful pedagogical experience – that is, one that they felt met their learning aims, was relevant for their stage of professional development and which provided skills that they could apply to their clinical practice – despite the potentially anxiety-provoking experience of healthcare simulation. We anticipated that the results would provide insights that lead to improved learner engagement and psychological safety in healthcare simulation, and hopefully healthcare education more broadly.

### Research questions

Our primary focus in this study was on students’ experiences during their simulation course – how they understood the causes and consequences of any anxious feelings, and how these impacted learning – addressed qualitatively via the second research question. We were also curious, however, given mixed findings in previous research, whether any clear correlation in our particular cohort would emerge between anxiety and simulation effectiveness. This was addressed quantitively in our first research question.

1. Does state anxiety before simulation correlate negatively with self-reported simulation effectiveness?
2. How do students experience the impact of their anxiety on learning during simulation?

## Methods

### Study design

This was a prospective observational study following a cohort of 5th year medical students from University College London who were undertaking the Essential Paediatrics Simulation course at Great Ormond Street Hospital (GOSH), with data collected before and after simulation. Undergraduate medical education at University College London follows a six-year Programme culminating in the receipt of the award Bachelor of Science (BSc) and Bachelor of Medicine and Bachelor of Surgery (MBBS). The curriculum consists of several themed modules running across each year integrated with a number of Clinical and Professional Practice modules interwoven throughout the whole degree. Years 1–2 lay the foundations for understanding the fundamentals of clinical science, followed by an Intercalated BSc in Year 3. In Years 4–6 a series of clinical placements across specialties link foundational knowledge to practice and aim to prepare students to thrive within clinical practice.

Recruitment for this study took place between 1^st^ October 2022 and 30^th^ January 2023. In accordance with the suggestions of the board approving the study, prospective students were sent an email from an administrator to their cohort letting them know about the option to be part of the study. After providing written, informed consent, students completed validated measure of state anxiety just before their course started. They then took part in the Essential Pediatrics Simulation Course. Following the course, they completed the Simulation Effectiveness Tool [19] immediately, and a semi-structured interview within 24 hours of the course. A mixed methods approach was used. Quantitative measures suited research question 1. Given this is a new area of research with contradictory quantitative findings, qualitative interviews provided the opportunity to triangulate and enrich quantitative data on anxiety, while also, addressing research question 2 by facilitating a more granular exploration of potential moderators of and contributors to anxiety, mindset and the subjective experience of simulation in this sample of students.

### Institutional approval

NHS Health Research Authority (HRA) approval was sought and obtained on 07 September 2022 (Project ID 314490, REC reference 22/HRA/3552).

### The Essential Paediatrics simulation course

By fifth-year, medical students would have all had some experience of simulation, likely lower fidelity than the Essential Paediatrics course. Medical Exams are also simulation-based, so they will have practiced for these. The Essential Paediatric course would likely differ from previous experiences in being higher fidelity and because the focus is not on passing or failing, as in an exam. Essential Paediatrics is a half-day course that runs throughout the academic year. Prior to the simulation aspect of the course, students take part in an interactive human factors session which covers behavioural and systems approaches to safety and quality in healthcare. Each session involves a pre-simulation briefing and introduction to staff and equipment. The pre-brief covers learning aims, expectations around simulation, acknowledgement of the fictional nature of simulation with discussion of what might help them to buy-in, along with requests for confidentiality and a reminder that they are not being assessed. The pre-brief might, for example, be the time when students mention previous experiences of simulation, or that simulation reminds them of medical exams, which would be discussed in the group (see Supplementary File 1 for pre-brief guide). Subsequently, in pairs, students take part in a simulated scenario where they are asked to manage an acute emergency in a child: one of sepsis, arrythmia or seizures. The scenario timeline is semi-structured, to be responsive to the interventions and management plans of the students. Following this, the students regroup to debrief on the scenario, along with the remaining students who observed the scenario from another room. The debrief covers technical skills (clinical knowledge and technique), non-technical skills (e.g., communication, teamwork, situational awareness, leadership), and emotive aspects of the simulation. (Supplementary File 1 contains further details on the course.)

### Data collection and analysis

#### Quantitative.

We collected baseline demographic data, including age and sex. In addition, we administered the following questionnaires:

1. The state questions on the State-Trait Anxiety Inventory (STAI): [20], made up of multiple questions on a 4-point Likert scale, responses are summed to get a discrete measure of anxiety. This is a common well-validated anxiety inventory used in equivalent types of research.
2. Simulation Effectiveness Tool [19]. This validated measure addressed our aim to examine participant’s perceptions of the simulation course, centering on key clinically relevant learning objectives, including effectiveness of simulation; how well learning objectives, e.g., in assessment and treatment were met; applicability to clinical practice; and confidence. These were measured via a questionnaire consisting of 3-point Likert scale questions.

Quantitative data was analyzed using SPSS 29 (IBM SPSS Statistics, New York). Numeric variables were presented using mean and standard deviation as data was normally distributed. The linear associations between numeric variables were presented using Pearson’s correlation coefficient. Linear regression models were used to quantify associations between STAI anxiety and simulation effectiveness score after controlling for confounders.

#### Sample size and power calculations.

Previous research from Gosselin [16] into the relationship between state anxiety and simulation effectiveness found a moderate correlation of r = 0.42 corresponding to a coefficient of determination (r-squared) of approximately 0.18. Setting the univariable coefficient of determination to be 0.15 (assuming a weaker relationship) in our study, we estimated that after accounting for anxiety and other sociodemographic factors, the coefficient of determination would be increased to 0.2. To detect a coefficient of determination of 20% with a power of 95% and type I error probability of 0.05, a sample size of 55 would be required. We expected a dropout rate of 20%, so we needed to recruit 66 participants to account for these possible missing observations. Preliminary data from participants showed a smaller effect size than anticipated and a greater ease in recruitment than anticipated, leading to a larger sample size target of approximately 80 (allowing for approximately 15–20% attrition).

#### Qualitative.

A subset of candidates also completed qualitative interviews. A semi-structured interview including broad open preliminary questions (‘How did you find the simulation?’) was designed with the aim of eliciting students’ experiences in a non-directive manner. In line with grounded theory, early interviews informed prompts for subsequent interviews [21]. Interviews took place either face to face in a confidential space at the hospital, or online via secure video call. Thematic analysis of interview data was informed by the principles outlined by Braun & Clark [22]:

1. Data immersion
2. Identifying initial codes
3. Identifying preliminary themes
4. Identifying relationships between themes
5. Identifying overarching themes
6. Revisiting previous stages to ensure themes are grounded in the data, with representative quotes being used in the final report.

No sample size was predetermined for qualitative data collection; instead, based on the information power doctrine [23] interviews continued until sufficient detail emerged to answer the research questions. Power was increased, and therefore required sample size decreased, by having clear and circumscribed research aims, studying a specific participant population (5^th^ year medical students), basing qualitative analysis on established grounded theory and thematic analysis methodologies, having trained interviewers who were able to establish rapport with candidates and increase the quality of the dialogue (GD is a clinical psychologist by background), and using an appropriate analysis strategy as detailed above. This process meant that most of the interviewed candidates were students who were recruited earlier in the study timeline; however, candidates in later phases of recruitment were also interviewed to ensure representativeness of themes across the set.

NS transcribed and identified initial codes. GD separately identified codes. Comparison and discussion was undertaken until a consensus was reached. Only subsequently were higher level themes examined. GD explored a subset of qualitative data for quality assurance and again highlight differences. Final themes were compared with original data as a further check of quality. Data was stored digitally on secure institutional servers, with access limited to the research team. The consolidated criteria for reporting qualitative research (COREQ) [24] checklist can be found in supplementary material, which includes quality assurance comments including on research reflexivity.

## Results

### Missing data

Of the 83 consented individuals, the data of 6 candidates failed to upload due to technical issues. Out of the 77 remaining participants in this study, 13% (n = 10) had at least one missing observation in age (1.3%), sex (2.6%), or simulation effectiveness score (11.7%), leaving us with 67 participants. Participants with missing data had similar baseline characteristics and outcome measures when compared to those without missing data. Therefore, it was assumed that missing values were missing completely at random (MCAR). In the statistical analysis, participants with missing data were removed using listwise deletion. Listwise deletion involves removing any participant with at least one missing value on any variable and this approach achieve unbiased results under the assumption of MCAR. Comparing regression analysis results based on list wise deletion and multiple imputation, we found that our analysis results were robust to the missing data.

### Descriptive statistics

**Fig 1:**
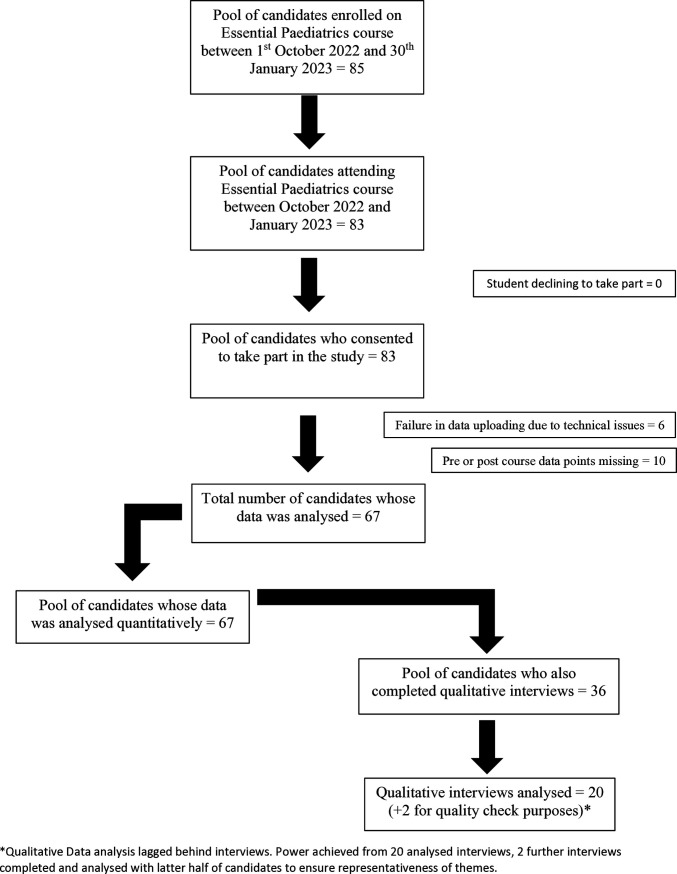
Flowchart of participants through study.

Fig 1 shows the flow of participants through the study. The average age of students who completed the simulation exercise was 22.9 years (SD 1.1). Of the 67 students included in the analysis, 65.7% (n = 44) were female, reflecting the current balance of students in the medical school cohort. The average score of students on the STAI was 35.1 (SD 9.8). This aligns with mean STAI scores for college students (36.5 for men, 38.8 for women) and is within the range of a normative population, below cut-off for clinically significant anxiety [25]. The mean score on simulation effectiveness was 17.9 (SD 2.7).

Regarding our first research question, there was no significant correlation between STAI and simulation effectiveness (p = .11; see Supplementary File 1 for further details). Our qualitative results offer a fine-grained exploration of how participants’ experienced their anxiety in relation to their learning on the course, the focus of our second research question.

### Qualitative themes

As Table 1 illustrates, three key overarching themes were identified. These are presented below with subthemes. These themes arose from participants’ reflections on their anxiety and elucidated how anxiety was inextricably linked to realism, meaning and learning in simulation.

**Table 1:** Overarching and sub- themes.

Anticipation of the unknown	Simulation as real	Being observed
Increasing cognitive load	Responsibilities	Exposure or scrutiny
Uncertainty not always aversive	Communication and teamworking	Observation as supportive
	Managing Leadership roles	Benign observation
	Assignment of roles	Critical self-assessment
	Real-time multi-tasking	Facilitating greater empathy
	Asking for help	Stance of the educators
	Contrast to exam/classroom	

#### Anticipation of the unknown.

At the beginning of their course, many participants reported a build-up of anxiety:

*I think the nerves were building up before and then they kind of peaked just before you step into the room. (Participant (p) 25)*

Some responded to this by trying to make the unknown more certain and felt reassured when more certain:

*My first thought was I need to know where all the stuff is, like what drawers contain what, and I also want to know like I want to guess what the station is. (p. 1)*

*I think going second was nice because we knew more or less what to expect, and, anyway, we had time to like think about what to do. (p. 17)*

As well as it being emotionally difficult to tolerate, uncertainty was seen by many participants as potentially **increasing cognitive load**.

*It’s like a new room, new setup. You just don’t really know where things are. (p. 5)*

The increase in anxiety also impaired the memory recall.

*One of the [candidates] came out after and basically said he knew the guidelines inside out, but he was kind of like stunned into silence – so the other person basically took control. (p. 3)*

The **uncertainty was not always viewed as aversive** as it offered an opportunity to learn:

*I think it’s good actually to go in with a kind of blank knowledge base… I think the areas I gained from most were things that I knew very little about. (p. 2)*

Many candidates linked this ‘blank knowledge base’ to what might be faced in real clinical practice, leading to our second overarching theme.

#### Simulation as real.

The realism of the simulation scenarios contributed to the anxiety for many participants:

*I felt as though I was a little bit out of my depth and nervous as to what the next steps would be, especially because it did feel quite real. The noises that were being made and the beeping and all of that and monitors on the screen, so it did feel a little bit overwhelming at times. (p. 13)*

It also set the simulation apart from other educational experiences, and elicited more spontaneous relational and emotive responses:

*Even though we do a lot of stuff in practice like in [a medical exam] situation, it is very different to being in a more realistic setting. (p. 3)*

*I feel like part of me was starting to get a little bit snappy; mostly because the situation had dramatically changed, one of the members of the team was on the phone, so he couldn’t communicate with all of us. (p. 1)*

These more emotive responses also tied to a realisation among the students of what might be required of them professionally:

*You’re like, “OK, yeah I do need to think about this” – and I think like when you’re studying in books and when you’re doing questions a lot of it is like “Oh that will be decisions that I’m going to have to make in the future,” but you don’t really take in and think about how that’s actually going to feel. I especially thought it was good that the nurse questioned me because that’s what’s going to happen. Having to think about what I was saying and back up my reasoning in like a time-pressured situation was really valuable. (p. 8)*

Several candidates reported that unlike previous classroom learning, simulation brought with it an insight into the potential **responsibilities** they would hold as a doctor. These included the challenges of sensitively **managing leadership roles**, something they had not previously covered in their training, and the thought of how others would perceive their attempts to step into the leadership role.

*So, then you’re pretending to be the decision-maker for the first time and then this person is going, “How would you like me to help you?” which is actually a very loaded question…you definitely don’t want to be like the most assertive person, you don’t want to be like: “No I want you to do this.” (p. 1)*

*I would hope that I’m not like the doctors that just talks and dictates what people should be doing, but I think I had also like been really conscious of not wanting to look silly in front of the nurse. (p. 8)*

The demands of **communication and teamworking** were commented on by many candidates as contributors to anxiety, but also as revealing professionally useful insights into their relational tendencies:

*I think, personally, I didn’t know when I should step forward or step back. Normally I just want to stand on the fence and let other people do it, but then, when it’s silence, sometimes you feel like you should step forward but you don’t know if anyone else will. (p. 24)*

The contrast between intellectual understanding and its application under pressure in a team setting also informed the **assignment of roles**, and students described the novel experience of having to adjust to working within a group:

*When it’s a group, let’s say three of you, one has to take a back seat and you follow someone else, so then you have to kind of match their way of thinking, which I found a little bit difficult because I remember things at my own pace. (p. 5)*

The students spoke of new insights into situational awareness: the nature of decision-making in real time, and the tension between this and the demands and realities of the situation.

*At one point I think we asked [the nurse] to do like two or three things at the same time and she’s just like, “Yeah, I’m doing this but there’s only one of me!” (p. 3)*

In the classroom or in exams, students have to verbalise or transcribe their decisions. Here, they had to navigate **real-time multi-tasking** within a clinical space where the time and resource required to prepare medicines, for example, had to be factored in:

*[The nurse said] “You’ve just said like four things that I can’t do at the same time” – so that was like one where you can think out loud but you have to differentiate when you’re asking people to do things, as opposed to thinking it. (p. 11)*

Several participants reported that they had not appreciated this reality previously, describing simulation as a stepping-stone to the real clinical world. Similarly, ***asking for help*** was mentioned by several students as a useful but stressful new skill that simulation exposed them to:

*After the first SIM, I was like, “You know what, this time we’re going to go in, we’re going to get the guideline book, we’ll call for help early.” (p. 28)*

In some cases, it wasn’t just that the simulated environment helped students realise the gulf between classroom recall and clinical acumen, some of the skills required were in direct **contrast to** that required in **an exam or classroom**. In the classroom or exam setting, for example, a student may be rewarded for their declarative knowledge, which is often imperfect and prone to attrition. In a real clinical setting it is often more effective to know where and how to find help, and which sources of information are reliable and which are not – abilities which drive the prime imperative of providing the right care at the right time. The habit of feeling that one needs to demonstrate knowledge above all else can be an impediment to optimal care.

*I feel like maybe passing you examines is not necessarily that useful for starting [as a doctor], like learning for [medical exams] I think is of limited use compared to examining or learning in a simulated environment. (p. 2)*

#### Being observed.

A related, though distinct, source of anxiety came from being observed by peers and facilitators. Participants reported a potential sense of **exposure or scrutiny** from the unavoidable fact of being observed.

*I did feel quite nervous, and so, then I was like, “OK, actually my skills are going to be exposed.” So maybe there is a bit of a personal imposter syndrome…I guess, and I should want to expose my skills and show the things I’ve learnt...to show I have learned something in four years in medical school. I enjoyed the exposure, I guess. (p. 15)*

Participants recognised that they too could *create* anxiety for their peers:

*[My peers] might feel criticised or judged and then it might make them feel worse about trying in the sim because, you don’t want to feel like someone’s watching you and telling you what you did wrong. (p. 4)*

Some linked this scrutiny to an awareness that their practice would be observed in real clinical work, thus circling back to the idea of professional **responsibility** and their identity as doctors. Several candidates recognised however, that observation leads to constructive criticism and feedback that could improve their skills:

*I found [the feedback] useful and it highlighted some things that I need to go over. (p. 24)*

Further, several candidates felt **observation could be supportive**, and that this could be aided by potentially assigning roles to observers. Perhaps the wish for observers to have roles was related to a more unnerving sense of scrutiny, elicited when the nature of the observation remained ambiguous. One candidate recognised this ambiguity as rife for projection of his own standards onto others:

*Seeing my colleagues do it as well, having known that I had also done it, was like: you know, it’s okay, we’re all learning here – but maybe if it was the other way around…had I seen the other group do it first and been like, “Oh, you should have done this and that,” and then when I couldn’t do it in the second round maybe I would have really felt: why am I not getting this straight or why am I not getting this right? (p. 8)*

Others appreciated that a difference may exist between self-reported performance and peer-reported performance, suggesting an awareness of an overly critical perspective:

*It was interesting to see the contrast between how it felt I’ve done and how [the group] saw us working together and being systematic. So it was interesting to see that actually those feelings might not be rooted in reality or rooted in logic. (p. 13)*

Thus the act of **benign observation** corrected an overly **critical self-assessment**. Further, being both observer and participant **facilitated greater empathy** for something that might appear easier from an observer role:

*We watched other people while they were doing it, and, when they were doing it, it actually seemed a lot easier from the outside; but, once you’re there you see how stressful it is. (p. 17)*

The most pedagogically useful sense of observation was the capacity to step back and reflect kindly but critically on one’s own practice. This key aim of simulation was described by several candidates:

*It was very interesting to see how my system kind of goes out the window initially when I’m faced with something [real], rather than faced with a textbook example… I think that’s something to reflect on, like how I could be better at managing pressure and just going through my normal process. (p. 19)*

Participants reported valuing the protected space for reflection in the debrief:

*When [in the debrief] we were reflecting, hearing opinions, it was really interesting because I’ve never really spoken to these people before. (p. 1)*

Participants commented that, though the expertise of the staff could make them feel nervous, the **stance of the educators** helped with the process of making their anxiety manageable and facilitating a more benign sense of observation:

*I think once I got out of the simulation I felt: “Oh, I messed up!”. During the debrief, I think just going through it you realise that you actually did a lot! (p. 17)*

Thus, educators appeared to be able, via their stance and instruction, to navigate some participants towards a more benign and pedagogically useful framing and experience of observation and anxiety during simulation.

## Discussion

We investigated whether anxiety negatively impacted perceived effectiveness of simulation. We found no significant association between state anxiety and simulation effectiveness. Instead, the qualitative data revealed a rich and nuanced relationship between anxiety and simulation experience. We found that anxiety was inextricably linked to meaning and motivation during the simulation scenario. This supports and expands on previous research into the mixed relationship between anxiety and learning [6,13–15] and has important clinical implications.

The psychological literature frames anxiety as a signal of threat [26,27]. The nature of this threat often depends on the way in which internal and external stimuli are interpreted. If a lion bursts through the door, the fight-flight response is instinctive, and there is little room for interpretation – there is a threat to life, and the reaction is immediate. A traffic jam also increases anxiety, but here the anxiety may signal an internal threat, for example, posed by one’s own rage, which can feel frightening. Anxiety over exams may arise because of the fear of failure, the threat of exclusion, and the shame that may arise from these. Anxiety may therefore not always be a primary emotion, but a signal, readying our body for some unknown future danger or challenge.

In healthcare simulation, the threats that elicited anxiety, reported by participants, were a fear of the unknown; the way in which the realism of the scenarios highlighted gaps in learning as well as pressures and responsibilities that they would face as medics; and the nature of observation, which ranged from scrutinous to benign. Reflecting on these elements provided students with new insights into the way they manage emotions, relationships, communication and cognitive loads in clinical emergencies. The educators were revealed to have a protective role. A supportive stance and clear session structure –– introductions, pre-brief, simulation, reflective debrief – helped students to feel safe and to value observation and feedback.

### Clinical implications

Anticipation and intolerance of uncertainty are well-known contributing factors to anxiety [28,29]. While uncertainty is inherent at the beginning of healthcare simulation, reducing extraneous uncertainty as far as possible while still maintaining curricular objectives may minimise detrimental impacts. Previous research indicates the benefits of a pre-brief for increasing psychological safety, reducing stress and optimizing learner outcomes [30]. Further, educators who normalise feeling of anxiety in relation to uncertainty can prevent a secondary sense of shame or embarrassment, which would likely exacerbate the negative aspects of anxiety. Previous research highlights the detrimental impact of extraneous cognitive load [14]. A thorough pre-brief, clear simulation plan and educator support reduces this load, which has been shown to improve simulation experience and effectiveness [31,32]. Students in our study reported that being placed in a realistic relational healthcare environment placed demands on their cognitive load that classroom or exam demands did not. They valued the opportunity that this realism provided for new learning, especially in the area of communication and team-working – realising, for example, that asking a team member to do four things at once was not possible, unlike memorising a four-item protocol for an exam. They found that seeking ways to reduce cognitive load allowed them to benefit optimally from this realistic environment, rather than becoming overwhelmed. Decreasing extraneous cognitive load may thus afford the greatest opportunity to take benefit from rather than be overwhelmed by this embodied and relational learning experience.

Students reported that following instructions from other students, asking for help, and adopting a leadership position caused anxiety because this placed students in a vulnerable position, which felt threatening. Historically these communicative or relational aspects of the role have been seen as non-essential “soft skills” in medical training, but many workplaces now place great emphasis on these skills. Exposure to incivility hinders clinical performance, affecting team working and patient care [33,34], and a growing body of research into moral injury [35] in healthcare workers speaks to the very real consequences of not having the opportunity to reflect upon the emotive and relational nature of the work. The implication is that learning activities which place the student in a real relational situation and encourage reflection on feeling and communication as well as clinical subject matter, are likely to be of benefit for their professional development. This is in keeping with recent recommendations from a meta-analysis to prioritise communication training in healthcare [36].

Medical students and doctors are traditionally expected to “know it all” – lengthy exams, high academic expectations, and quick-fire questioning of juniors on ward rounds all feed into the perception that recall under pressure is the hallmark of a true doctor. Yet, this can exacerbate unhelpful tendencies such as a need to know, a fear of uncertainty, and an internalising of shaming experiences as being unavoidable during learning. Recent research has called for a need to address medicine’s ‘culture of perfection’ [37], highlighting that maladaptive perfectionism – high concern over making mistakes, setting unrealistic standards and goals with constant comparison to others increases psychological distress [38] and a sense of ‘imposter syndrome’ which can, at its worst, translate into increased risk for physician suicide [37]. Demonstrating knowledge of the core curriculum is obviously necessary but our study suggests that a more well-rounded form of competence includes realising that successful performance in real clinical settings requires asking for help quickly, recognising demands on and limitations of resources, and being able to navigate uncertain environments. The latter were reported as novel learning experiences by participants in our study.

Further, participants reflected on how harshly they can judge themselves and their colleagues, but it appeared that with support they could find a more generous and supportive perspective. Educational and pastoral interventions that normalise anxiety while supporting students to place emphasis on a capacity to tolerate the unknown, to engage in civility and to recognise that they can inform and sculpt medical culture, are likely to be more useful than interventions that seek solely to impart medical expertise, or those that *tell* rather than *show* about psychological safety. Being told to be respectful at work is one thing, noticing that under threat you become judgemental and panicked, which impacts on your capacity to be respectful, is another – the latter is the beginning of a developmental process of self-reflection. Previous research has found that increasing self-compassion mediates link between perfectionism and depression in undergraduates, and maladaptive perfectionism has been suggested as a target for intervention [38,39]. In the current study, students reported a favourable experience of the non-judgemental and accepting stance of educators.

### Research implications

The items on the state measure of the STAI mainly comprise straightforward descriptors of current state, with an absence of interpretative statements. One could hypothesise that STAI items such as “strained,” “tense,” “nervous,” “jittery” do not distinguish between embodied changes caused by readying oneself in a helpful way for the real demands of an upcoming task, and embodied changes caused by an anticipation of shame due to a maladaptive sense of perfection, and a projection of impending judgement onto one’s peers. These nuanced differences in the nature of anxiety may inform the complex association with performance found in previous research and may indicate benefits in future research of going beyond a study of anxiety to an exploration of underlying and causal factors, particularly those related to the interpretation or meaning of the upcoming learning event. Our study is hopefully a reminder that qualitative methods can reveal important data which quantitative factors that are too tightly defined a priori may not elucidate. The value of studying anxiety as a factor in relation to learning, without an unpacking of contributors to and consequences of anxiety is worthy of further critical reflection.

### Limitations

The chief limitation of this study is that inferences beyond the particular participant group studied must be made cautiously. Instead, the study might be viewed as a generator of further hypotheses to be tested in larger studies. A further limitation in the design of the study relates to the point at which state anxiety was measured. Students completed the STAI before the simulation pre-brief. The purpose of the pre-brief is to contain and reduce anxiety – as such, it is difficult to infer whether the lack of relationship between anxiety and simulation effectiveness might have partly been due to a successful pre-brief. A measure of state anxiety before and after the pre-brief, and indeed after the simulation, would be an important addition to future studies. It may also be beneficial to randomise some participants to receive a pre-brief while others are in the control group.

### Conclusions

The reasons for, interpretations of and responses to anxiety, including by educators and other peers, were more important than the presence of anxiety for participants in the current study. These reasons for anxiety can include beliefs that students carry about the perceived impact of their not knowing or asserting themselves, as well as the assumptions they make about what others are thinking when observing them. Raising awareness of such thoughts can help students re-appraise them. Further, the study hopefully demonstrates the value of qualitative explorations of anxiety and stress alongside quantitative. Asking an intelligent participant what they are worried about can helpfully enrich quantitative or chemical measures of anxiety and stress.

## Supporting information

S1 File Supplemental document file. Includes participant information sheet, informed consent form, questionnaire scales, details of the Essential Paediatrics Course, COREQ checklist and link to publicly available dataset.
(DOCX)
