# Correction: “My skills are going to be exposed” – Anxiety, meaning and professional identity during simulation-based learning in medical students: A mixed method study

**DOI:** 10.1371/journal.pone.0335536

**Published:** 2025-10-28

**Authors:** Gareth Drake, Niki Skaltsa, Kritika Kalia, Chibueze Ogbonnaya, Asha Padhiar, Samuel Morrish, Pratheeban Nambyiah

The fifth author’s name is spelled incorrectly. The correct name is: Asha Padhiar. The correct citation is: Drake G, Skaltsa N, Kalia K, Ogbonnaya C, Padhiar A, Morrish S, et al. (2025) “My skills are going to be exposed” – Anxiety, meaning and professional identity during simulation-based learning in medical students: A mixed method study. PLoS One 20(7): e0327306. https://doi.org/10.1371/journal.pone.0327306.

The seventh author, Pratheeban Nambyiah, should also be noted as the corresponding author. Pratheeban Nambyiah’s email address is: p.nambyiah@ucl.ac.uk.
