# Supplementary material for: “My skills are going to be exposed” – Anxiety, meaning and professional identity during simulation-based learning in medical students: A mixed method study
Source: PLoS One. 2025 Jul 9;20(7):e0327306. doi: 10.1371/journal.pone.0327306 (PMC12240371; doi:10.1371/journal.pone.0327306)
Supplement: S1 File — Includes participant information sheet, informed consent form, questionnaire scales, details of the Essential Paediatrics Course, COREQ checklist and link to publicly available dataset. (DOCX) [file pone.0327306.s001.docx]

**Supplementary Information File**

Participant Information sheet page 2

Consent form page 5

State-Trait Anxiety Inventory page 6

Dweck Mindset Growth Scale page 8

Simulation Effectiveness Tool page 9

The Essential Paediatrics Course page 10

The GOSH pre-brief tool page 11

COREQ page 12

Quantitative Results Tables page 16

Link to data on Kaggle page 18

**Participant Information Sheet**

**Medical students’ attitudes towards paediatric clinical simulation**

**Invitation**

You are being invited to take part in this research project. Prior to this, we* would like to provide with adequate information to ensure you understand why the research is being done and what it will involve. Please take the time to read through the following information. If there is anything that is not clear or if you have any questions, please do ask us.

**What is the purpose of this project?**

The research aims to explore among medical students, the relationships between attitudes towards paediatric simulation, previous simulation experience, anxiety levels and a measure of growth mindset (the way challenges and set-backs are viewed).

The results of the study will hopefully inform simulation educators’ approach to pre-briefs and debriefs with the aim of increasing engagements with simulation and psychological safety for learners.

**What will happen?**

If you agree to be in the study, you will be asked to complete a questionnaire that will ask about your mood, anxieties, about the way you view set-backs and challenges, and about your experiences of simulation, which we estimate will take roughly 10-15 minutes. In addition, you will be asked to take part in a semi-structured interview before and after your simulation session, which we expect to last for 20-30 minutes. These interviews will be held virtually via zoom or face to face (dependent on what is feasible and convenient). These interviews will be recorded to refer back to for data analysis.

Data will be stored anonymously, anything you say will be entirely separate from your placement, will not be reported back or count towards an assessment of your placement, and will not affect anything in relation to your placement at Great Ormond Street Hospital. If you drop-out and do not wish any of your data to be used in the study it will be immediately deleted.

The results of the study will be written up in a research paper, which will hopefully be published in a relevant journal. Direct quotes may be used in the final research article but with no identifiable information. The research may yield future studies in the same area, and the data may therefore be used again, for example, to build a case for a larger study, or in a related study into the experiences of simulation candidates. If this is the case, again, any data used will remain completely anonymous, and no identifiable data will be passed on to other researchers.

**hereafter referring to the study sponsor, Great Ormond Street Children’s Hospital Foundation Trust*

**What is my role in this?**

You will be asked to answer questions during the interviews and on the questionnaire openly and honestly. There will be no other commitments or restrictions associated with participating.

**Risks and Benefits**

Participating in this research in not anticipated to cause any significant disadvantage or distress. The greatest burden will be your time, and the accompanying flow chart will hopefully make clear the time requirements in the study. You will be asked about your anxiety levels. If you are required to travel into GOSH on any day you wouldn’t be attending for the Essential Paediatrics Course, you will be reimbursed for this travel (please speak to a member of the research team about this). If you report significant anxiety levels, we can assist you, if you would like, with signposting to appropriate services and resources. The main benefit is to the future experience of staff at GOSH, and hopefully as a result, GOSH patients. You may gain some personal benefit from an extended opportunity to reflect on your educational experience on the Essential Paediatric Simulation Course, though this is by no means a guarantee or aim of the study.

**What if I change my mind?**

You are free to withdraw at any time without giving a reason. Your details will be deleted from our records and we will not contact you any further for the purposes of this research. Your decision to withdraw from the research will not have any effect on the educational opportunities available to you.

### How will we use information about you?

We will need to use information from you for this research project.

This information will include your initials, contact details, age and gender. People will use this information to do the research or to check your records to make sure that the research is being done properly. People who do not need to know who you are will not be able to see your name or contact details. Your data will have a code number instead. We will keep all information about you safe and secure.  Once we have finished the study, we will keep some of the data so we can check the results. We will write our reports in a way that no-one can work out that you took part in the study. You can opt to have your personal contact information (email address) retained by the research team in order to receive study results

### What are your choices about how your information is used?

- You can stop being part of the study at any time, without giving a reason, but we will keep information about you that we already have.

Where can you find out more about how your information is used?

You can find out more about how we use your information

- by asking one of the research team
- by sending an email to research.governance@gosh.nhs.uk
- or by speaking to our data protection officer:[**your.data@gosh.nhs.uk**](mailto:your.data@gosh.nhs.uk)

**Who has reviewed this study?**

This study has been reviewed and approved internally by the sponsor via Great Ormond Street Hospital’s Clinical Research Adoptions Committee (CRAC) and externally by the Health Research Authority.

**What if I want to find out more?**

You can discuss this with the researchers, or request to contact Principal Investigator Pratheeban Nambyiah. If something goes wrong or you are unhappy with any aspect of the research process you also may wish to contact someone outside of the immediate research group, such as the GOSH Freedom to Speak-Up Guardian (speakup@gosh.nhs.uk) or the GOSH Research Governance Team (research.governance@gosh.nhs.uk). Please don’t hesitate to ask for any of these contact details.

**Research Consent Form**

**Medical students’ attitudes towards paediatric clinical simulation**

**Please tick within the box to agree with the following statements**

- I confirm that I have read and understood the information about the above titled project, provided in the participant information.
- I confirm I have had the opportunity to ask questions and the researchers have answered these to my satisfaction.
- I understand that any information recorded during the project will remain confidential and will only be used for research purposes only. No information will be made available to the public for any reason.
- I understand that data collected during the study, may be looked at by individuals from regulatory authorities or from the NHS Trust, where it is relevant to my taking part in this research.
- I understand that the information collected about me may be used to support
  other research in the future and may be shared anonymously with other researchers.
- I understand my participation is voluntary and I am able to withdraw myself from the project at any time.
- I consent to the use of my data in research, publication and archiving as explained in the participant information.
- I consent to audio interviews being recorded as part of the project.
- I agree to take part in the above study.

I consent to my contact details being retained in order to receive a results summary of the study Yes / No (You can say no to this point of consent and still take part in the research).

………………………………………………………………………. ……………………………………………………………………

Name of Participant Signature

Date:

…………………………………………………………………….. ……………………………………………………………………

Name of Researcher Signature

Date

**State Trait Anxiety Inventory**

Read each statement and select the appropriate response to indicate how you feel

right now, that is, at this very moment. There are no right or wrong answers. Do not

spend too much time on any one statement but give the answer which seems to

describe your present feelings best.

**1 2 3 4**

**Not at all A little Somewhat Very Much So**

1. I feel calm 1 2 3 4

2. I feel secure 1 2 3 4

3. I feel tense 1 2 3 4

4. I feel strained 1 2 3 4

5. I feel at ease 1 2 3 4

6. I feel upset 1 2 3 4

7. I am presently worrying

over possible misfortunes 1 2 3 4

8. I feel satisfied 1 2 3 4

9. I feel frightened 1 2 3 4

10. I feel uncomfortable 1 2 3 4

11. I feel self confident 1 2 3 4

12. I feel nervous 1 2 3 4

13. I feel jittery 1 2 3 4

14. I feel indecisive 1 2 3 4

15. I am relaxed 1 2 3 4

16. I feel content 1 2 3 4

17. I am worried 1 2 3 4

18. I feel confused 1 2 3 4

19. I feel steady 1 2 3 4

20. I feel pleasant 1 2 3 4

**References:**

**Background:**

The STAI is a validated 20 item self report assessment device which includes separate

measures of state and trait anxiety. The original STAI form was constructed by Charles

D. Spielberger, Richard L. Gorsuch, and Robert E. Lushene in 1964. The STAI has been

adapted in more than 30 languages for cross-cultural research and clinical practice (Sesti,

2000). Various reliability and validity tests have been conducted on the STAI and have

provided sufficient evidence that the STAI is an appropriate and adequate measure for

studying anxiety in research and clinical settings (Sesti, 2000). McIntrye, McIntyre, and

Silverio (in press) validated the STAI for Portuguese communities. Several items on the

STAI were reversed coded (Items 1, 2, 5, 8, 11, 15, 16, 19, 20).

Recommended for studying anxiety in research and clinical settings.

**Developers:**

Charles D. Spielberger, Richard L. Gorsuch, and Robert E. Lushene in 1964

**Copyright:**

Consulting Psychologists Press, Inc.

**Reliability:**

The stability of the STAI scales was assessed on male and female samples of high school

and college students for test-retest intervals ranging from one hour to 104 days. The

magnitude of the reliability coefficients decreased as a function of interval length. For the

Trait-anxiety scale the coefficients ranged from .65 to .86, whereas the range for the

State-anxiety scale was .16 to .62. This low level of stability for the State-anxiety scale is

expected since responses to the items on this scale are thought to reflect the influence of

whatever transient situational factors exist at the time of testing.

**Assessment:**

Spielberger, C. D. (1972). *Anxiety: Current trends in theory and research: I.* New York, N.Y.:

Academic Press.

Spielberger, C. D. (1980). *Test Anxiety Inventory. Preliminary professional manual*. Palo Alto, CA:

Consulting Psychologists Press.

Spielberger, C. D. (1983). *Manual for the State-Trait Anxiety Inventory (STAI)*. PaloAlto, CA:

Consulting Psychologists Press.

Download this page as a PDF file

**Dweck mindset scale**

**Scoring:**

To calculate the total score for each participant, take the average rating of the items by adding respondents’ answers to each item and dividing this sum by the total number of items (3).

**Sources:**

Dweck, C. S. (1999). *Self-theories: Their role in motivation, personality, and development.* Philadelphia: Psychology Press.

Dweck, C. S., Chiu, C. Y., & Hong, Y. Y. (1995). Implicit theories and their role in judgments and reactions: A world from two perspectives. *Psychological Inquiry, 6*(4), 267-285.

**Instructions:** Read each sentence below and then circle the *one* number that shows how much you agree with it. There are no right or wrong answers.

1. You have a certain amount of intelligence, and you can’t really do much to change it.

| Strongly agree | Agree | Mostly agree | Mostly disagree | Disagree | Strongly disagree |
| --- | --- | --- | --- | --- | --- |
|  |  |  |  |  |  |

2. Your intelligence is something about you that you can’t change very much.

| Strongly agree | Agree | Mostly agree | Mostly disagree | Disagree | Strongly disagree |
| --- | --- | --- | --- | --- | --- |
|  |  |  |  |  |  |

3. You can learn new things, but you can’t really change your basic intelligence.

| Strongly agree | Agree | Mostly agree | Mostly disagree | Disagree | Strongly disagree |
| --- | --- | --- | --- | --- | --- |
|  |  |  |  |  |  |


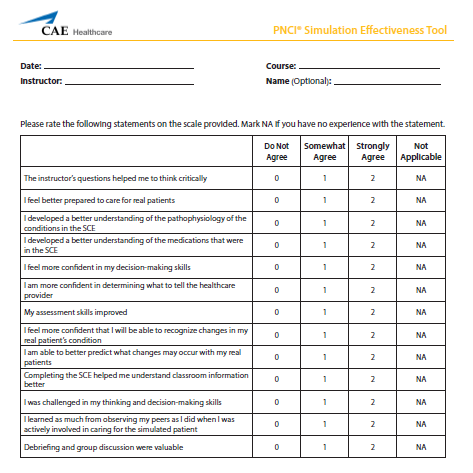


**Further Details about the Essential Paediatrics Course**

- The course is half day (Mondays, 12:30 - 5pm) and forms part of the student's one week placement in paediatric at GOSH
- It is not graded
- It is a required part of their placement
- Each student participates in one scenario and observes the rest. There are typically 3-4 scenarios in each half day session, depending on the size of the group.
  - The scenarios typically last 10 minutes depending on how the team progresses.
  - They typically centre around a topic covered within APLS training (advanced paediatric life support). We reassure students that there will never be an instance where the patient does not have a pulse or dies as the aim of the simulation is not to go through basic life support algorithms but to expand their clinical reasoning, knowledge and introduce the importance of human factors.
- The manikins are chosen based on the scenario. We use: Paediatric HAL ® (Gaumard) and Simbaby (Laerdal).
- The debrief typically last 30 minutes. The person doing the debrief is de-brief trained and their peers are pat of the debrief when they are observers.
- The human factors section is an interactive taught session, lasting 30 minutes, at the beginning of the session. The concepts learnt are then integrated into each debrief that follows.


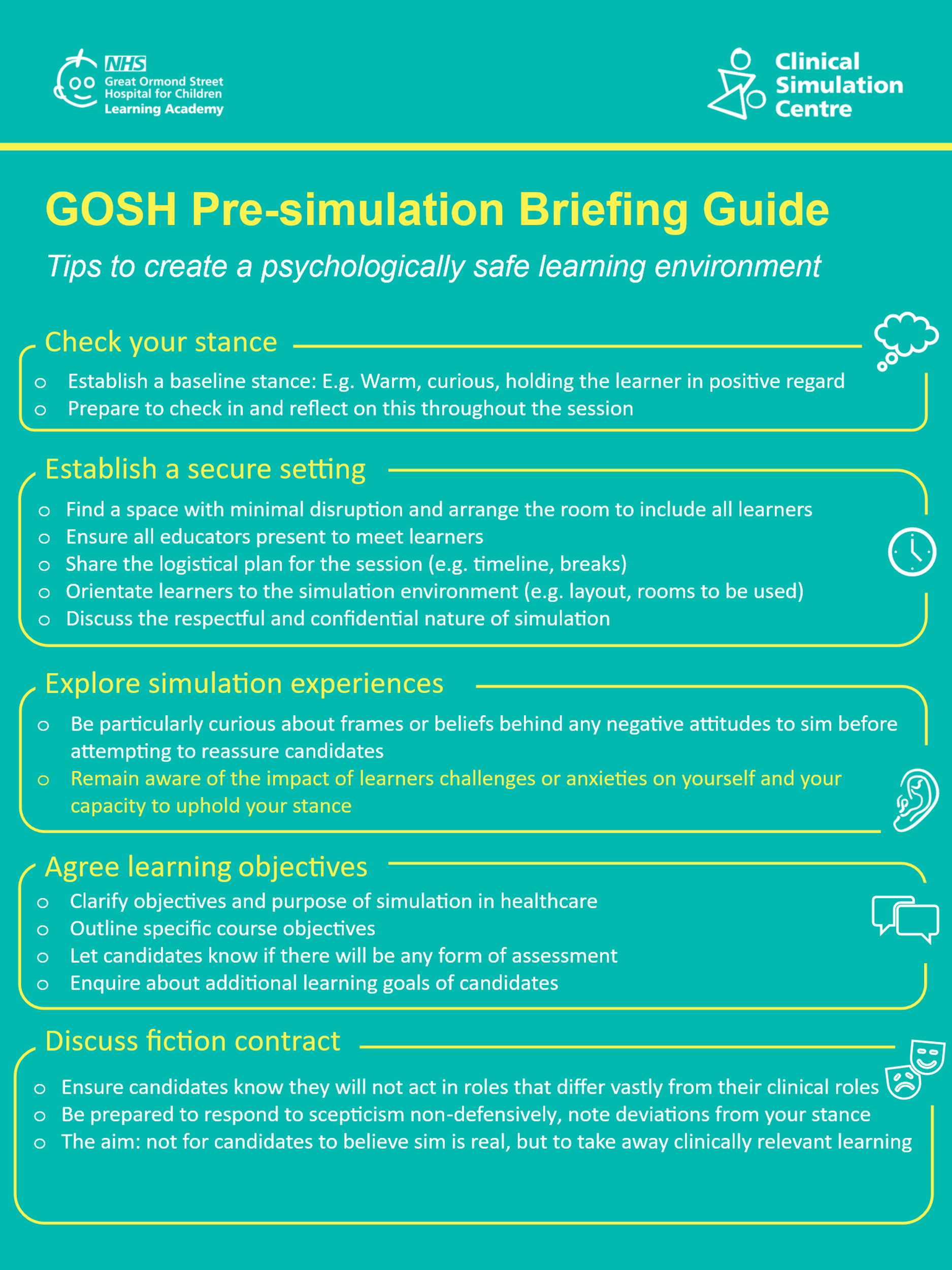


From: Drake, G., & Drewek, K. (2024). “I Hate Sim!”—Using Psychotherapeutic Concepts to Help Educators Attend to Challenging States of Mind During Simulation Prebriefs. *Simulation in Healthcare*, 10-1097

**Consolidated criteria for reporting qualitative research (COREQ)**

Below, our qualitative methodology and results are mapped to the *Consolidated criteria for reporting qualitative research* (COREQ) checklist

1. Interviewer/facilitator: Kritika Kalia (KK) & Niki Skaltsa (NS) conduced the interviews under the supervision of Gareth Drake (GD)

2. Credentials: KK & NS are medical fellows, GD is a Clinical Psychologist

3. Occupation: All were completing the above roles in the Clinical Simulation department at Great Ormond Street Children’s Hospital

4. Gender: KK & NS are women, GD is a man

5. Experience and training: GD is a trained researcher with a doctoral level knowledge of qualitative methods and experience supervising students. KK & NS had several sessions prior to study commencement with recommended reading, practice of interviewing and regular discussions, along with regular supportive contact throughout.

6. Relationship established: NS and KK knew participants prior to the study as a contact point and link to the simulation course at Great Ormond Street. It was ensured that first contact was via an administrator who held no power over potential candidates to reduce as far as possible a sense of pressure to participate. NS and KK taught on the simulation course alternately. It was ensured that whoever taught a participating student did not also conduct the research interview with that student. The supportive approach to students taken throughout teaching by all faculty, as well as the existing relationships with researchers, may have helped participants feel psychologically safe to share more openly during interviews. Conversely it may have made it more difficult to speak critically of their experience. The themes appear to include elucidating insights that are not overly preoccupied with relations with researchers, and which corroborate existing research into anxiety, performance and learning.

7. Participant knowledge of the interviewer: The participants knew the interviews in relation to their student placements and teachings at Great Ormond Street Hospital.

8. Interviewer characteristics: KK & NS introduced themselves in the beginning of each interview and explained study’s goals and objectives. Their relationship to the students is made explicit above.

9. Methodological orientation and theory: Elements of Grounded Theory alongside Thematic Analysis were used.

10. Sampling: Convenience.

11. Method of approach: An email invitation to participate in the study was sent via an administrator to the cohort to let them know of the study.

12. Sample size: 36 in a larger study of 67

13. Non-participation: No participants refused, approx. 10% quantitative data lost due to internet signal issues.

14. Setting of data collection: Zoom/MS Team or face-to-face in private room.

15. Presence of non-participants: No.

16. Description of sample: Students completing a paediatric simulation course at a children’s hospital

17. Interview guide: The questionnaire prompts were designed by GD based on the research aims. They were pilot tested on simulation centre staff. Initially they comprised the following: *How did you find that? Did it match your expectations? How did it differ? What aspects did you find challenging? What aspects were unforeseen? How did you find the pre-brief? How did you find the simulation itself? How did you find the debrief? How did you find the stance of the educators? Were you anxious? Did you have any other strong emotions? What helped you tolerate these? Did anything make it worse?* The interviews were semi-structured and iteratively altered depending on initial themes and responses. Iterative qualitative interviews that are semi-structured evolve as they go, as such the exact prompts in each interview varied. If one participant stated, “I found being observed difficult”, an interviewer may say, “In what way?” If another participant doen’t mention being observed, the interviewer may notice this and say, “What about being observed (as it was a theme for all other ppts)”?

18. Repeat interviews: N/A.

19. Audio/visual recording: Recordings were audio-recorded then stored encrypted until transcribed.

20. Field notes: N/A.

21. Duration: Interviews took approx. 15 minutes.

22. Data saturation: Saturation was discussed alongside the complementary concept of data power (Malterud, 2016) where sufficient interviews continued to answer reliably the research question

23. Transcripts returned: No.

24. Number of data coders: Two, NS & GD

25. Description of the coding tree: NS initially identified a list of codes that developed into minor themes. GD independently found similar themes in 3 separate interviews. Discussion led to a list of key minor themes. GD then subsumed these into 3 major themes. These are all indicated in the results section.

26. Derivation of themes: themes derived from the data

27. Software: N/A

28. Participant checking report: Participants didn’t provide feedback on findings

29. Quotations presented: Quotations were included throughout the results from a range of participants.

30. Data and findings consistent. Raw data was consistent with existing literature, overall themes, and quantitative data.

31. Clarity of major themes. 3 major themes relating to the realism of simulation, the unknown, and the nature of observation overarched the subsumed minor themes.

32. Clarity of minor themes. Minor themes and discrepancies in relation to a major theme (e.g. observation as supportive versus observation as scrutinising) are presented.

**Quantitative Results**

The focus of the main body of this paper is on the qualitative results as we demonstrated that in our cohort, anxiety and simulation effectiveness were not correlated. We were interested initially in a potential moderating factor – Growth Mindset – a well-researched concept relating to how individuals view set-backs. This concept proved redundant in our study, as we found no correlations, precluding the need to examine moderating factors – nevertheless, the 3-item questionnaire that students completed formed part of our quantitative analysis and is therefore included below.

**Table 1: Bivariate correlation (p-value) for numeric variables.**

|  | **Age** | **STAI** | **Growth Mindset** | **SE** |
| --- | --- | --- | --- | --- |
| **Age** | 1 | -0.051 (0.683) | 0.175 (0.130) | -0.026 (0.837) |
| **STAI** |  | 1 | -0.053 (0.648) | -0.118 (0.343) |
| **Growth Mindset** |  |  | 1 | 0.109 (0.377) |
| **SIM** |  |  |  | 1 |

n.b. Pearson correlation coefficients are reported as parametric assumptions met.

A linear regression model was fitted to understand the relationship between simulation effectiveness and state anxiety after controlling for growth mindset, age, and sex (Table 2). STAI had a weak negative non-significant association with simulation effectiveness score, even after accounting for age, sex, and growth mindset. There were no significant associations between the other predictors (sex, growth mindset and age) and simulation effectiveness.

**Table 2: Linear regression for the association between simulation effectiveness and age, sex, STAI and growth mindset.**

| **Predictor Variable** | **Coefficient** | **95% CI** | **P-value** |
| --- | --- | --- | --- |
| Age | -0.075 | (-0.700, 0.550) | 0.812 |
| Sex=Female | -1.154 | (-2.571, 0.263) | 0.108 |
| STAI Total | -0.027 | (-0.101, 0.046) | 0.458 |
| GM Average | 0.257 | (-0.369, 0.884) | 0.415 |

These results support our first null hypothesis – that there is no significant association between state anxiety and simulation effectiveness – and renders invalid our second research question (does growth mindset mitigate the negative effect of state anxiety on simulation effectiveness).

Dweck C. What having a “growth mindset” actually means. Harvard business review. 2016 Jan 13;13(2):2-5.

Dweck C. Mindset-updated edition: Changing the way you think to fulfil your potential. Hachette UK; 2017 Jan 12.

**Link to data on Kaggle**

[Paediatric clinical simulation](https://www.kaggle.com/datasets/garethdrake/paediatric-clinical-simulation)
